# Comparative studies on the pathogenicity and tissue distribution of three virulence variants of classical swine fever virus, two field isolates and one vaccine strain, with special regard to immunohistochemical investigations

**DOI:** 10.1186/1751-0147-50-34

**Published:** 2008-09-05

**Authors:** Katinka Belák, Frank Koenen, Hans Vanderhallen, Christian Mittelholzer, Francesco Feliziani, Gian Mario De Mia, Sándor Belák

**Affiliations:** 1 Department of Virology, National Veterinary Institute, S-751 89, Uppsala, Sweden; 2 Department of Pathology and Wildlife Diseases, National Veterinary Institute, S-751 89, Uppsala, Sweden; 3 Department of Virology, Epizootic Swine Virology, CODA-CERVA, Groeselenberg 99, B-1180 Ukkel, Belgium; 4 Istituto Zooprofilattico Sperimentale dell'Umbria e delle Marche, via G Salvemini 1, 06126, Perugia, Italy; 5 Institute of Structural Biology, Biozentrum, University of Basel, Klingelbergstrasse 70, CH-4056 Basel, Switzerland; 6 Joint Research and Development Division, Departments of Virology, National Veterinary Institute and Swedish University of Agricultural Sciences, S-751 89, Uppsala, Sweden

## Abstract

**Background:**

The aim of this study was to compare the tissue distribution and pathogenicity of three virulence variants of classical swine fever virus (CSFV) and to investigate the applicability of various conventional diagnostic procedures.

**Methods:**

64 pigs were divided into three groups and infected with the highly virulent isolate ISS/60, the moderately virulent isolate Wingene'93 and the live attenuated vaccine strain Riems, respectively. Clinical signs, gross and histopathological changes were compared in relation to time elapsed post infection. Virus spread in various organs was followed by virus isolation, by immunohistochemistry, applying monoclonal antibodies in a two-step method and by *in situ *hybridisation using a digoxigenin-labelled riboprobe.

**Results:**

The tissue distribution data are discussed in details, analyzing the results of the various diagnostic approaches. The comparative studies revealed remarkable differences in the onset of clinical signs as well as in the development of the macro- and microscopical changes, and in the tissue distribution of CSFV in the three experimental groups.

**Conclusion:**

The present study demonstrates that in the case of highly and moderately virulent virus variants the virulence does not affect the pattern of the viral spread, however, it influences the outcome, the duration and the intensity of the disease. Immunohistochemistry has the advantage to allow the rapid detection and localisation of the virus, especially in cases of early infection, when clinical signs are still absent. Compared to virus isolation, the advantage of this method is that no cell culture facilities are required. Thus, immunohistochemistry provides simple and sensitive tools for the prompt detection of newly emerging variants of CSFV, including the viruses of very mild virulence.

## Background

Classical swine fever (CSF) is a highly contagious viral disease of swine and wild boars, causing severe economic losses mainly in countries with dense pig populations. The causative agent is classical swine fever virus (CSFV), a small enveloped, positive-stranded RNA virus that belongs to the genus *Pestivirus *in the *Flaviviridae *family [1,2]. The genus also comprises bovine viral diarrhoea virus (BVDV) and border disease virus (BDV) of sheep.

Although CSF has been known for more than 150 years, the losses to this disease are still extremely high. For example the 1997–98 outbreaks of CSF caused very heavy losses in the Netherlands, when approximately 12 million pigs were lost due to the disease (about 700,000 heads), culling and welfare reasons [3].

A number of observations show that antigenic variations exist among CSFV strains and the various field isolates can vary considerably in virulence. Highly virulent viruses cause peracute or acute forms of the disease with high morbidity and mortality in pigs, irrespective of age and breed. In contrast, viruses of moderate to low virulence may cause a very mild or inapparent disease. In the last three-four decades, the most common clinical picture of CSF has changed from acute to subacute, chronic or inapparent forms [4,5]. These changes in the clinical manifestation of the disease frequently complicate the early detection and proper diagnosis of the CSF, considering that the very mild clinical symptoms might easily be overlooked. The delayed diagnosis may cause uncontrolled spread of CSF and heavy losses in large swine populations. Considering this situation, there is a high need to perform comparative studies on the tissue distribution of various variants of the virus in order to study virus biology and to assure the diagnosis.

The diagnosis can be complicated by the uncharacteristic profiles of CSF clinical symptoms, which may lead to delayed identification of new outbreaks (see World Organisation for Animal Health, OIE, ). A further diagnostic problem is that rather poor information is available concerning the pathogenicity and invasion capacity of various virulence variants of CSFV. The early studies on viral pathogenicity and invasion were restricted to single strains and comparative aspects were not discussed. For example, Ressang [6,7] described the quantitative distribution of the virulent Brescia strain in various tissues in increasing intervals. Subsequently, the studies were extended to involve comparative analysis of more than one strain. Such work was performed by Kamolsiriprichaiporn et al. [8] who compared the pathogenicity of the virulent Weybridge and of the low virulent New South Wales strains. Japanese researchers performed comparative immunohistochemical studies on organ specimens of pigs infected with the highly virulent ALD strain or with the less virulent Kanagawa 74 strain, respectively [9].

The aim of this study was: i) to gain further knowledge on the tissue distribution and pathogenicity of CSFV, by directly comparing the *in vivo *effects of three virulence variants of the virus; ii) to investigate the applicability of various diagnostic procedures to detect the various virulence variants in the experimentally infected host animals. For these purposes, the virus distribution was determined by virus isolation (VI), the CSFV antigen was visualised in paraformaldehyde fixed, paraffin-embedded tissue sections by a monoclonal antibody based, two-step immunohistochemical method and the viral RNA was detected by *in situ *hybridisation, using a digoxigenin (DIG)-labelled riboprobe.

By comparing the spread of three virulence variants of the virus in 64 animals, this study was performed in order to examine the tissue distribution of CSFV in the natural host, to obtain data of comparative pathology and to compare the applicability of virus detection methods. These observations will contribute to a better understanding of the viral pathogenesis and to the introduction of more effective measures to control CSF.

## Methods

### Viruses and animals

The studies involved three virulence variants of CSFV. The highly virulent ISS/60 virus was isolated from an Italian landrace pig, while the moderately virulent Lorraine isolate, alias Wingene'93, originated from a Belgian domestic pig herd [5]. The attenuated vaccine strain Riems [subgroup 1.1., 10] was used as an avirulent representative of CSFV. The isolates were checked to be free from African swine fever virus (ASFV) by using haemabsorption-inhibition test as well as ELISA. The presence of BVDV was excluded by virus isolation and by immunoperoxidase tests (IPX) using monoclonal antibodies. All assays were performed according to OIE guidelines (In: Manual of standards for diagnostic tests and vaccines. Ed 5. Chap 2.1.12. Paris: OIE, 2004; Office International des Epizooties/World Organization for Animal Health).

To compare the virulence variants, 67 conventional weaner hybrid pigs (20–25 kgs body mass) were used. The animals were clinically healthy on arrival and serologically tested to be free of CSFV, BVDV, porcine reproductive and respiratory syndrome virus (PRRSV), encephalomyocarditis virus (EMCV) and Aujeszky's disease virus (ADV) by using the standard diagnostic procedures of our institutes and our routine serological tests [11].

### Experimental design

A standardised protocol was used for the animal experiments, carried out by two partners of EU research project FAIR PL 95–707 in Belgium (Experiment/group II) and in Italy (Experiments/groups I and III). The conditions were harmonised within the consortium of the project. Animal experiments were approved by the ethical committees in both countries. Upon arrival, the animals were clinically examined, randomly numbered and housed in completely separated high-security isolation units. Experiments I and II involved 25 pigs each, while Experiment III was composed of 17 animals.

After 6-days acclimatisation the animals (24-24-16) were intranasally inoculated with 2 ml volumes of the viruses (10^3 ^TCID^50 ^per/ml) as follows: group I with ISS/60, group II with Wingene'93 and group III with Riems. In each experiment one uninfected, separately housed pig was used as negative control.

The pigs were sequentially killed by electrocution on various post infection days (PIDs) as indicated in Tables 1 and 2.

**Table 1:** Results of Experiment I; pigs, infected with the highly virulent isolate, ISS/60

**PID(h)**	**Pig No.**	**Tonsils**	**Spleen**	**Kidneys**	**Ln. 1**	**Ln. 2**	**Ln. 3**	**Lungs**	**Heart**	**Cerebr.**	**Cerebel.**	**Musc. 1**	**Musc. 2**
**-1**	**1**	*-/-	-/-	-/-	-/-	-/-	-/-	-/-	-/-	-/-	-/-	-/-	-/-
**12 h**	**2**	-/-	-/-	-/-	-/-	-/-	-/-	-/-	-/-	-/-	-/-	-/-	-/-
	**3**	-/-	-/-	-/-	-/-	-/-	-/-	-/-	-/-	-/	-/-	-/-	-/-
**1**	**4**	-/-	-/-	-/-	-/-	-/-	-/-	-/-	-/-	-/-	-/-	-/-	-/-
	**5**	-/-	-/-	-/-	-/-	-/-	-/-	-/-	-/-	-/-	-/-	-/	-/-
	**6**	-/-	-/-	-/-	-/-	-/-	-/-	-/-	-/-	-/-	-/-	-/-	-/-
	**7**	-/-	-/-	-/-	-/-	-/-	-/-	-/-	-/-	-/-	-/-	-/-	-/-
**2**	**8**	+/-	+/-	-/-	+/-	-/-	-/-	-/-	+/-	-/-	-/-	-/-	-/-
	**9**	+/-	-/-	-/-	-/-	-/-	-/-	-/-	-/-	-/-	-/-	-/-	-/-
	**10**	+/+	-/-	-/-	+/+	-/+	-/+	-/-	-/-	-/-	-/-	-/-	-/-
	**11**	+/+	-/-	-/-	+/+	+/+	++	-/-	-/-	-/-	-/-	-/-	-/-
**3**	**12**	+/+	+/+	+/+	+/+	+/+	+/+	+/-	+/-	+/-	+/-	-/-	-/-
	**13**	+/++	+/-	-/-	+/+	+/+	+/+	-/-	-/-	-/-	-/-	-/-	-/-
**4**	**14**	+/+++	+/+	+/+	+/+++	+/++	+/+++	+/++	-/-	-/-	-/-	+/-	-/-
	**15**	+/+++	+/+++	+/+++	+/+++	+/+++	+/+++	+/-	+/-	+/-	+/-	+/-	+/-
**5**	**16**	+/+++	+/+++	+/+++	+/+++	+/+++	+/+++	+/-	+/-	+/-	+/-	-/-	-/-
	**17**	+/+++	+/+++	+/+++	+/+++	+/+++	+/+++	+/+	+/-	-/-	-/-	+/-	+/-
	**18 !**	+/n	+/n	+/n	+/n	+/n	+/n	+/n	-/n	+/n	+/n	-/n	-/n
	**19 !**	+/n	+/n	+/n	+/n	+/n	+/n	+/n	+/n	-/n	-/n	+/n	+/n
**6**	**20 !**	+/n	+/n	+/n	+/n	+/n	+/n	+/n	+/n	+/n	+/n	-/n	-/n
	**21 !**	+/n	+/n	+/n	+/n	+/n	+/n	+/n	-/n	-/n	-/n	-/n	-/n
	**22 !**	+/n	+/n	+/n	+/n	+/n	+/n	+/n	+/n	+/n	+/n	+/n	+/n
	**23 !**	+/n	+/n	+/n	+/n	+/n	+/n	+/n	+/n	+/n	+/n	+/n	+/n
**7**	**24 !**	+/n	+/n	+/n	+/n	+/n	+/n	+/n	+/n	+/n	-/n	-/n	-/n
**8**	**25**	-/+++	+/+++	+/+	+/+++	+/+++	+/+++	+/+++	+/-	+/+	+/++	-/-	+/-

*Results of virus isolation/immunohistochemistry.

Severity of reactions: as measured by IHC = - = negative, + = 1–3 foci/section, ++ = 4–10 foci/section, +++ > 10 foci/section.

Ln. 1 = ileocecal lymph node

Ln. 2 = mesenteric lymph node

Ln. 3 = submandibular lymph node

Musc. 1 = M. longissimus dorsi

Musc. 2 = M. quadriceps

PID = post infection day.

! = found dead.

n = not available.

h = hours

**Table 2:** Results of Experiment II; pigs, infected with the moderately virulent isolate, Wingene'93

**PID**	**Pig No.**	**Tonsils**	**Spleen**	**Kidneys**	**Ln. 1**	**Ln. 2**	**Ln. 3**	**Lungs**	**Heart**	**Cerebr.**	**Cerebel.**	**Musc. 1**	**Musc. 2**
**0**	**1 (25)**	*-/-	-/-	-/-	-/-	-/-	-/-	-/-	-/-	-/-	-/-	-/-	-/-
**1**	**2 (1)**	-/-	-/-	-/-	-/-	-/-	-/-	-/-	-/-	-/-	-/-	-/-	-/-
	**3 (2)**	-/-	-/-	-/-	-/-	-/-	-/-	-/-	-/-	-/-	-/-	-/-	-/-
**2**	**4 (3)**	-/-	-/-	-/-	-/-	-/-	-/-	-/-	-/-	-/-	-/-	-/-	-/-
	**5 (4)**	-/-	-/-	-/-	-/-	-/-	-/-	-/-	-/-	-/-	-/-	-/-	-/-
**3**	**6 (5)**	-/-	-/-	-/-	-/-	-/-	-/-	-/-	-/-	-/-	-/-	-/-	-/-
	**7 (6)**	-/-	-/-	-/-	-/-	-/-	-/-	-/-	-/-	-/-	-/-	-/-	-/-
**4**	**8 (7)**	-/-	-/-	-/-	-/-	-/-	-/-	-/-	-/-	-/-	-/-	-/-	-/-
	**9 (9)**	+/+	-/+	-/-	+/-	-/-	-/-	-/+	-/-	-/-	-/-	-/-	-/-
**5**	**10 (8)**	+/+++	-/-	-/-	+/-	+/-	+/++	-/+	-/-	+/-	+/-	-/-	-/-
	**11 (10)**	+/+-++	-/+++	-/-	+/++	+/+	+/+++	-/+	-/-	-/-	-/-	-/-	-/-
**6**	**12 (11)**	+/+	-/+++	-/-	+/+++	+/-	+/+++	+/+	+/-	-/-	-/-	-/-	-/-
	**13 (12)**	+/+++	+/+	-/-	+/-	+/++	+/+++	+/+	-/-	-/-	-/-	-/-	-/-
**7**	**14 (13)**	+/+++	+/+++	+/-	+/+++	+/+	+/+++	+/-	-/-	-/-	-/-	-/-	-/-
	**15 (14)**	+/+++	+/++	+/-	+/+	+/+	+/+++	+/+	-/-	-/nc	-/nc	-/-	-/-
**8**	**16 (15)**	+/++	+/+++	+/-	+/+	+/+	-/+++	+/-	+/-	-/nc	-/nc	-/-	-/-
	**17 (16)**	+/+++	+/+++	+/-	+/+++	+/++	+/+++	+/++	+/-	-/nc	+/nc	+/-	+/-
	**18 (17)**	+/+++	+/+++	+/-	+/+++	+/+++	+/+++	+/+++	+/-	+/-	+/-	+/-	-/-
**10**	**19 (18)**	+/++	+/+++	+/+	+/+	+/+++	+/+	+/++	+/-	+/-	+/-	+/-	-/-
	**20 (20)!**	+/+++	+/+	+/+	+/+	+/+	+/+++	+/+++	-/-	-/-	+/-	-/-	-/-
**12**	**21 (19)**	+/++	+/++	+/-	+/+	+/++	+/+	+/-	+/-	+/-	+/-	+/-	+/-
	**22 (21)**	+/+++	-/++	+/-	+/-	+/+	+/+	+/++	+/-	+/-	-/-	+/-	+/-
**14**	**23 (22)**	+/++	-/+	+/-	+/+	+/+	+/+	+/-	-/-	+/-	-/-	+/-	+/-
	**24 (23)!**	+/-	+/++	+/+++	+/+++	+/+++	+/+++	+/+++	-/-	-/-	-/-	+/-	-/-
	**25 (24)!**	+/+++	+/++	+/+	+/++	+/-	+/++	+/+	+/-	+/-	-/-	+/-	+/-

*Results of virus isolation/immunohistochemistry.

Severity of reactions: as measured by IHC = - = negative, + = 1–3 foci/section, ++ = 4–10 foci/section, +++ > 10 foci/section.

Ln. 1 = ileocecal lymph node

Ln. 2 = mesenteric lymph node

Ln. 3 = submandibular lymph node

Musc. 1 = M. longissimus dorsi

Musc. 2 = M. quadriceps

PID = post infection day.

! = found dead.

nc = not conclusive

### Clinical examinations and sample collection

The pigs were monitored daily for clinical signs. Rectal temperatures were recorded every day throughout the experiments. Blood samples were collected for VI on all sampling days.

After euthanasia or death, necropsies were performed and gross lesions were recorded. Tissue samples of tonsils, spleen, ileocoecal, mesenteric and submandibular lymph nodes, kidneys, lungs, heart muscle, cerebrum, cerebellum and striated muscle (M. longissimus dorsi and M. quadriceps) were collected from all animals except seven pigs, which died in Experiment I between PIDs 5 and 7.

### Virus isolation (VI)

VI was performed from tissue and blood samples. About 1 cm^3 ^of tissue samples were homogenised in 9 ml MEM culture medium using an Ultraturrax (Junke and Kunkel). The suspension was centrifuged at 4,000 × g for 10 min and 300 μl of the supernatant was inoculated onto a non-confluent monolayer of BVDV-free PK15 cell cultures on multi-dish plates (Falcon 35; 3047). Concerning the blood samples, serum was separated, 100 μl was diluted in 900 μl culture medium and 300 μl amount of the dilution was inoculated onto a non-confluent monolayer of BVDV-free PK15 cell cultures in a multi-dish plate. The plates were incubated for 48 hours, fixed with isopropanol and stained with a polyclonal immunoperoxidase conjugated polyclonal serum with a dilution of 120 (OIE Manuals 2004 and 2008, ).

### Direct immunofluorescence (DIF) in Experiment II

Due to practical reasons and the various technical facilities available at our institutes, the DIF studies were restricted to the 25 animals of experimental group II. This group was selected for the DIF investigations, considering that the moderately virulent (or low virulent) variants of CSFV have large epidemiological importance, since due to the lack of typical clinical manifestation these cases may easily be overlooked in the field. Considering that this may lead to a delayed detection of the disease, special attention should be focused on the comparative pathology of such variants of CSFV. The cell cultures or the cryostat sections were fixed with acetone and stained with fluorescent anti-CSF polyclonal serum by following OIE guidelines (In: Manual of standards for diagnostic tests and vaccines. Ed 5. Chap 2.1.12. Paris: OIE, 2004).

### Histopathology, immunohistochemistry (IHC) and *in situ *hybridisation (ISH)

For histopathological and immunohistochemical examinations, the collected tissue samples were fixed in 4%, freshly prepared, buffered paraformaldehyde, embedded in paraffin according to routine histological procedures and sectioned at the thickness of 5 μm. The sections were stained with haematoxylin-eosin for histopathological evaluation.

For immunohistochemical examinations, monoclonal antibody "WH 303", specific to CSFV glycoprotein E2, was kindly provided by Dr. David Paton, Veterinary Laboratory Agencies, Addlestone, (recent affiliation: Pirbright Laboratory), UK. The antibody was applied by a two-step peroxidase method, using the DakoEnVision +HP mouse Kit (Dakopatts, Glosstrup, Denmark). Briefly, deparaffinised tissue sections were rinsed in 0.5-mol/l Tris-HCl buffer, pH 7.6 containing 0.15 mol/l NaCl (TBS). Endogenous peroxidase was inactivated by incubating the sections with 1% (v/v) hydrogen peroxide in TBS for 20 min. The tissue sections were then rinsed thoroughly in TBS and incubated for a further 10 min with 2% bovine serum albumin (BSA) in TBS at room temperature before incubation with the primary antibody overnight at 4°C. The monoclonal antibody WH 303 was diluted 1: 200 in TBS containing 1% BSA. As negative controls, duplicate sections were incubated with 2% BSA instead of specific primary antibodies. The sections were washed three times for 5 min each time in TBS followed by 30 min incubation with one drop of peroxidase conjugated rabbit anti-mouse secondary antibody. After a washing step in TBS, peroxidase activity was visualised by incubation sections in TBS containing 0.06% (w/v) 3, 3'diaminobenzidine tetrahydrochloride (DAB, Sigma, St. Louis, USA) and 0.034% (v/v) hydrogen peroxide for 8 min. Finally, the sections were rinsed in tap water, counterstained in Mayer's haematoxylin and mounted with Entellan (Merck, Darmstadt, Germany).

*In situ *hybridisation was performed on sections processed as for IHC and mounted onto 3-aminopropyltrietoxysilane-coated slides (Sigma, St. Louis, MO, USA). Prior to deparaffinisation and rehydration in graded ethanol the slides were heated to 75°C for 15 min. In order to improve the probe penetration, the sections were digested with protease VIII 0.25 mg ml^-1 ^at 25°C for 15 min. Finally the slides were washed twice in distilled water, dehydrated in graded ethanol and air-dried. The DIG-labelled riboprobe was synthesised from a HindIII-BamHI fragment of an infectious cDNA clone of CSFV Riems cloned into pBlueScript II SK+ (Stratagene, La Jolla, CA). Negative strand RNA representing nucleotides 6436-5711 of the Riems full-length sequence was in vitro transcribed using the DIG RNA labelling kit (Roche). The hybridisation mixture consisted of 50% formamide, 10% dextran-sulphate, 2 × SSC (1 × SSC = 0.15 M sodium chloride, 0.015 M sodium citrate), 0.1 mM EDTA, 1 mM Tris-HCl pH 7.5, denatured salmon sperm DNA to a final concentration of 4 mg ml^-1 ^and 0.5 ng/μl freshly denatured DIG-labelled riboprobe. Sixty μl of the hybridisation mixture was applied per slide. The tissue sections were covered and sealed by Frame-Seal chambers (MJ Research Inc, Watertown, MA, USA). The slides were then placed into a PTC 200 Peltier Thermal Cycler (MJ Research Inc) equipped with an interchangeable Twin Towers *in situ *block, heated to 65°C for 15 min. The hybridisation was carried out at 55°C for 2 hours. A bovine herpesvirus type 5 (BHV-5) specific DIG-labelled probe [12] was used on duplicate sections as negative controls. After hybridisation the slides were gently washed as follows: twice (5 min each) with 4 × SSC at room temperature, twice (5 min each) with 1 × SSC at room temperature and once with 0.1 × SSC for 15 min at 55°C. The sections were not allowed to dry at any time during or following the washing steps of post-hybridisation. For the immunological detection of the digoxigenin-labelled hybrids, a DIG Nucleic Acid Detection Kit (Boehringer Mannheim, Germany) was used according to the manufacturer's instructions, utilizing an antibody-conjugate (anti-digoxigenin alkaline phosphatase conjugate, anti-DIG-AP) and an enzyme-catalysed colour reaction with 5-bromo-4-chloro-3-indolyl phosphate (BCIP) and nitroblue tetrazolium salt (NBT), providing a blue-coloured precipitate.

## Results

### Clinical signs and viraemia

In Experiment I (highly virulent virus), three pigs developed febrile reaction (40–40.3°C) at post infection day (PID) 1. From PID 2, twelve out of 18 animals showed pyrexia up to 42°C, which persisted throughout the observation period. Some pigs developed inappetence, apathy and mild diarrhoea from PID 1. Starting from PID 3, three animals showed staggering, shivering and incoordination. At PID 5, one piglet developed posterior paresis. At PID 8, the remaining one piglet showed nervous symptoms such as locomotoric ataxia and paresis. Cutaneous lesions were constantly absent. The animals, which were not sacrificed, died from PIDs 5 to 7 (Table 1). Viraemia, as recorded by virus detection in the serum samples, started at PID 2 in three animals and at PID 3 all the animals but two became viraemic, as it was shown by the VI assays. From PID 4 all pigs showed viraemia until the end of the experiment.

In Experiment II (moderately virulent virus) the first febrile reactions were noticed in two pigs at PID 2 and half of the inoculated animals successively developed fever, up to 41.5°C during the observation period. Apathy and inappetence were recorded at PID 11. At PID 12 diarrhoea and a stringent respiration were noticed. Skin haemorrhages and ataxia appeared one day before death, on PIDs 9 and 13. The animals that were not sacrificed died at PIDs 10 and 14 (Table 2). Viraemia started at PID 5 in one animal.

In Experiment III (avirulent vaccine strain), all the animals showed slightly elevated temperature with an average of 0.4 – 0.9°C from PID 1 until the end of the experiment. No other clinical signs were recorded in the group infected with the avirulent CSFV strain. Viraemia was not observed in this group.

### Gross pathology

In Experiment I, one animal presented a distinct swelling of the submandibular lymph nodes at PID 2. Spleen infarction was seen in one pig at PID 3. From PID 4, all the remaining animals showed evidence of typical CSF lesions characterized by severe enlargement of lymph nodes with haemorrhages in the periphery, spleen infarction and petechial haemorrhages in the renal cortex.

The macroscopic lesions in Experiment II were swollen lymph nodes with discrete petechial haemorrhages and haemorrhages in the kidneys of the pigs that were killed at PID 8. Only in the pigs sacrificed and died at the terminal phase of the experiment from PID 12 became the signs more pathognomonic.

At the post mortem examination of the pigs in Experiment III a general swelling of the lymph nodes was observed in one pig at 36 hours after inoculation. Mild haemorrhages were seen in the lymph nodes of the head and neck regions in one animal at PID 2.

No macroscopic pathological changes were observed in the uninfected control pigs.

### Virus isolation (VI) from tissue samples

The virus was detected (re-isolated) from the tissue samples in all the three experiments.

In Experiment I, the virus was isolated from the tonsils, spleen, lymph nodes and heart muscle at PID 2. Subsequently, the VI tests detected the virus from the tonsils and lymph nodes of all infected animals, with the exception of the tonsil samples of one pig (Table 1). The virus was also re-isolated from the spleen, kidneys, lungs, heart, brain and striated muscles, as shown in Table 1.

In Experiment II, CSFV was detected at PID 4 in the tonsil and ileocoecal lymph node of one pig. From PID 5, the virus was isolated from the tonsils and lymph nodes of all infected animals. VI detected the virus also in the spleen, kidneys, lungs, heart, brain and in the striated muscles, see Table 2.

In Experiment III, CSFV was isolated only from the tonsils of three animals at PIDs 3, 5 and 7 and from the ileocoecal lymph node of one pig at PID 7 and mesenteric lymph node of one animal at PID 8.

The results of virus isolation from tissue specimens are summarised in Tables 1 and 2.

### Direct immunofluorescence in Experiment II

By the means of DIF, the virus was detected in tonsils, in the superficial and crypt epithelial cells, macrophages, lymphoid and endothelial cells from PID 4 and the fluorescence staining remained fairly homogenous until PID 14, at the end of the experiment. In the spleen, immunofluorescence was first observed at PID 7 in lymphoid and endothelial cells. In the lungs, positive staining was found in the bronchiolar mucosal epithelial cells as well as in the alveolar macrophages and in a few endothelial cells from PID 8 until the end of the experiment. In the kidneys, only a small amount of positively stained duct epithelial, endothelial and mononuclear cells were observed in seven animals from PID 6. In the myocardium, immunostaining was seen only in one pig at PID 10. The immunoreactivity was observed in the endothelial cells of the small capillaries. In the brain and muscle specimens positive immunofluorescence staining has not been detected.

### Histopathological, immunohistochemical examinations and in situ hybridisation

Microscopic lesions were observed in the examined organs of pigs in all the three infected groups. The changes were more frequent and severe in Experiments I and II. The monoclonal antibody, specific to gp E2 of CSFV, gave specific positive cytoplasmic staining reaction in tonsils, spleen, lymph nodes, lungs and kidneys but not in myocardium and striated muscles. Further immunopositivity was detected in nervous tissues in one single animal in Experiment I (Tables 1 and 2).

#### Experiment I

**Figure 1:**
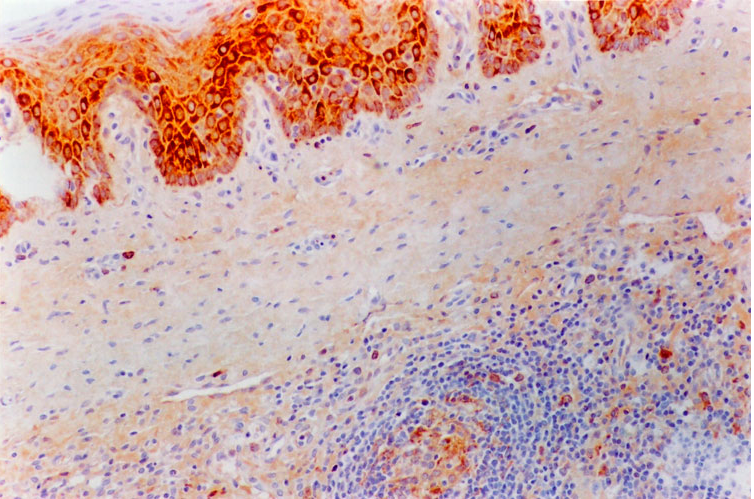
**Tonsil. Experiment I, PID 8. Positive immunohistochemical staining**. Tonsil. Experiment I, PID 8. Superficial-epithelial cells, macrophages and lymphoid cells staining intensely for CSFV antigen in the cytoplasm with a monoclonal antibody specific for glycoprotein E2 (WH 303). Immunohistochemistry; EnVision™ +HP mouse system. Magnification 540×.

In tonsils the lesions consisted of some cystically enlarged or plugged tonsillar crypts with cellular debris, neutrophil granulocytes and keratin. A mild hypertrophy of the follicles was observed from 36 hours after inoculation. Necrotic changes were also seen from PID 1. Specific immunoreactivity was detected first in a few crypt-epithelial cells and many migrating macrophages, as well as in the lymphoid cells at PID 2. The immunostaining became more disseminated from PID 4 in the crypt-epithelial cells, macrophages lymphoid and endothelial cells and remained fairly homogenous until PID 8, at the end of the experiment. In addition, at PID 8 very strong immunostaining was observed in the superficial-epithelial cells (Figure 1). In lymph nodes, a mild depletion/atrophy of the follicles was seen between 12–24 hours after inoculation, followed by a mild follicular and perifollicular hypertrophy from 36 hours after infection until the end of the experiment. Necrotic changes were seen first in the follicles at PID 1 and became then more diffuse. Acute focal haemorrhages were found in two lymph nodes. Specific immunostaining was observed in reticular cells, macrophages, lymphoid and a few endothelial cells from 60 hours after infection. A fairly uniform, lower amount of virus antigen could be detected in all the lymph nodes at PID 3 and a still uniform but higher amount of positively stained cells between PID 4 and 8. In spleen, a mild depletion/atrophy of the follicles/periarterial lymphatic sheaths (PALS) and perifollicular hyperplasia was observed 12 hours after inoculation, followed by a mild hypertrophy. Immunoreactivity was first observed at PID 3 in reticular cells, macrophages, lymphoid and endothelial cells. In kidneys, six pigs had a very mild focal mononuclear interstitial nephritis between 12 hours and 2 days after inoculation. Only a small number of positively stained duct epithelial, endothelial and mononuclear cells were observed in one animal at PID 8. In lungs, very mild non-suppurative bronchointerstitial inflammatory changes were observed in all the pigs. These lesions were considered as non-specific. Specific immunoreactivity was found in the bronchial and bronchiolar mucosal epithelial cells, in the alveolar macrophages and in a few endothelial cells from PID 4 in two animals. In heart muscle, specific histopathological changes were not observed. In cerebrum and cerebellum, the main changes were confined to the vessels in form of vasculitis consisting of infiltration of mononuclear cells into the wall and around the small blood vessels, most frequently in meninges and white matter. In many cases, swelling and degenerative changes of the endothelial cells occurred. In some cases the vascular changes were accompanied by focal gliosis. The lesions developed one day after inoculation. Positive immunostaining was detected in one single animal at PID 8. In muscles, very mild focal acute muscle degeneration to variable degree and oedema were observed in all the three infected groups and control animals, throughout the experiment (results not repeated below).

#### Experiment II

**Figure 2:**
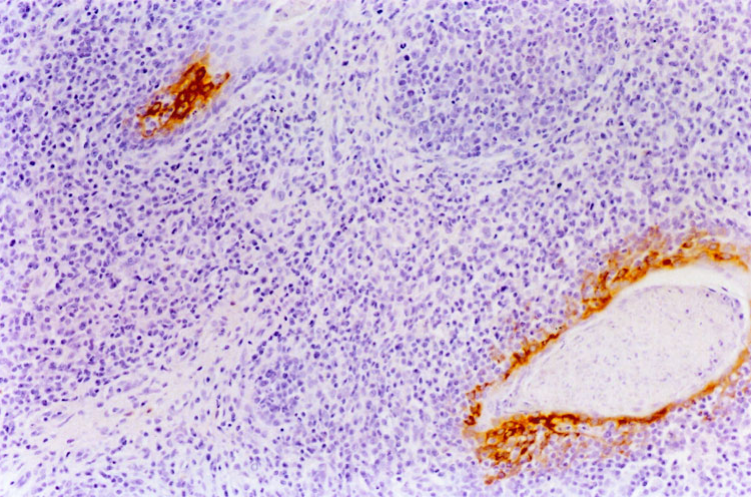
**Tonsil. Experiment II, PID 7. Positive immunohistochemical staining**. Tonsil. Experiment II, PID 7. Immunoreactivity to WH 303 monoclonal antibody as a cytoplasmic rim in the crypt-epithelial cells. Immunohistochemistry; EnVision™ +HP mouse system. Magnification 540×.

**Figure 3:**
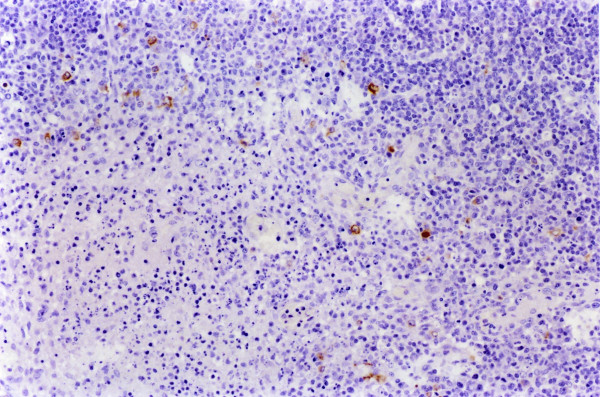
**Lymph node. Experiment II, PID 6. Positive immunohistochemical staining**. Lymph node. Experiment II, PID 6. Immunoreactivity to WH 303 monoclonal antibody in the cytoplasm of the reticulocytes and macrophages. Immunohistochemistry; EnVision™ +HP mouse system. Magnification 540×.

**Figure 4:**
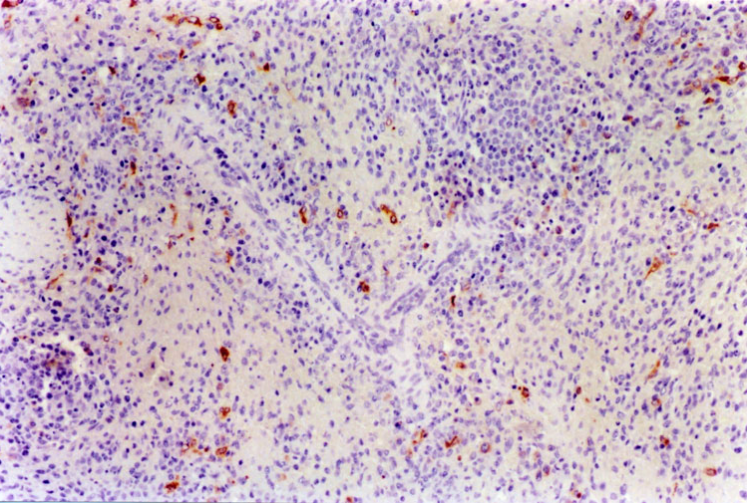
**Spleen. Experiment II, PID 7. Positive immunohistochemical staining**. Spleen. Experiment II, PID 7. Immunoreactivity to WH 303 monoclonal antibody in the cytoplasm of reticulocytes and macrophages. Immunohistochemistry; EnVision™ +HP mouse system. Magnification 540×.

**Figure 5:**
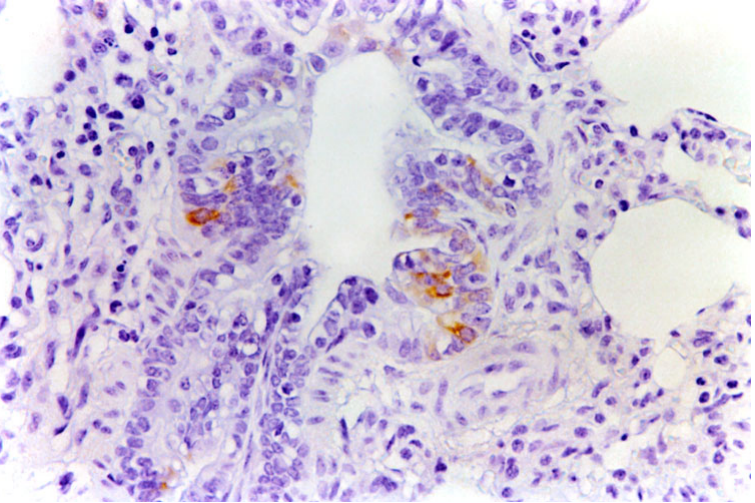
**Lungs. Experiment II, PID 14. Positive immunohistochemical staining**. Lungs. Experiment II, PID 14. Immunoreactivity to WH 303 monoclonal antibody in the cytoplasm of the bronchiolar epithelial cells. Immunohistochemistry; EnVision™ +HP mouse system. Magnification 1080×.

**Figure 6:**
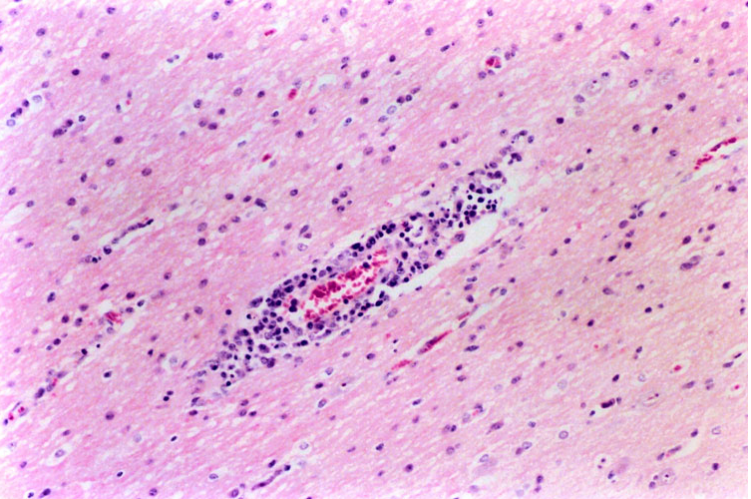
**Brain, blood vessel. Experiment II, PID 12. Degenerative changes**. Brain, blood vessel. Experiment II, PID 12. Degenerative changes (pyknosis and karyorrhexis) of the endothelial cells. Haematoxylin-eosin staining. Magnification 540×.

In tonsils, the microscopic lesions consisted of some cystically enlarged or plugged tonsillar crypts with cellular debris, neutrophil granulocytes and keratin. Necrotic changes were seen from PID 6 and became very severe in the two pigs, which died at PID 14. Specific immunoreactivity was first detected exclusively in the crypt-epithelial cells, from PID 4. From PID 7 a higher amount of virus antigen was detected in the crypt-epithelial cells, migrating macrophages and lymphoid cells as well as in endothelial cells (Figure 2). From PID 10 the viral antigen was detected even in the superficial-epithelial cells. The immunostaining remained fairly homogenous until PID 14, at the end of the experiment. In lymph nodes, a mild depletion/atrophy of the follicles was observed from PID 7 in six pigs and a mild follicular as well as perifollicular hypertrophy from PID 3, respectively PID 6 in 12 respectively 3 pigs until the end of the experiment. These changes were most evident in the submandibular lymph nodes. Acute focal haemorrhages were seen in the submandibular lymph node of seven animals from PID 5. Follicular necrosis was observed at PID 5 and 6 in the submandibular lymph node. From PID 7 more diffuse necrotic changes were seen occasionally in all the three examined lymph nodes. Specific immunostaining was observed in reticular cells, macrophages, lymphoid and a few endothelial cells from PID 5. Immunoreactive macrophages and lymphoid cells were most evident in the reactive centre of the follicles. In the submandibular lymph node a greater number of positively stained cells were observed than in the ileocoecal and mesenteric lymph nodes between PID 5 and 8 (Figure 3). After that, a fairly uniform but lower amount of virus antigen could be detected in all the lymph nodes until PID 14. In spleen, mild follicular/PALS atrophy was recorded from PID 3. Focal haemorrhages were observed from PID 7 as well as necrotic lesions mainly in the white pulp from PID 4. These necrotic changes were very severe and characterized as vascular necrosis in one pig and as an acute-subacute fibrinopurulent-necrotic peritonitis in another one, which died at PID 14. Specific immunoreactivity was first observed at PID 4 in reticular cells, macrophages, lymphoid and endothelial cells (Figure 4). In kidneys, a few acute focal haemorrhages were seen, mainly in the medulla, in five animals from PID 6. Furthermore, mild focal mononuclear interstitial nephritis was observed in four animals and a mild acute focal glomerulonephrosis was detected in two animals at PID 14. In one pig, which died at PID 12, acute pyelonephritis was observed. Only a small amount of positively stained duct epithelial, endothelial and mononuclear cells were observed in four animals from PID 10. In the lungs, very mild non-suppurative bronchointerstitial inflammatory changes were observed in five pigs from PID 10. They consisted of vascular lesions with fibrinoid necrosis and tendency to thrombus formation. In one of these animals focal acute fibrinotic pneumonia with necrosis was also seen at PID 14. Immunoreactivity was found in the bronchial and bronchiolar epithelial cells, in the alveolar macrophages and in a few endothelial cells from PID 4 (Figure 5) until the end of the experiment. In the hearts, the pathological findings were confined to the smaller vessels of the myocardium in three pigs, from PID 10. In one animal, which died at PID 10, a marked endothelial proliferation was observed. Necrotic vasculitis occurred in two pigs, which died at PID 14. Specific immunostaining was not detected. In the cerebrums and cerebellums, similar vasculitis was observed as in Experiment I, with severe degenerative changes (Figure 6) from PID 10 until the end of the experiment in almost all pigs. In some cases the vascular lesions were accompanied by focal gliosis. In two cases, mild endothelial proliferation was observed at PID 10 and 14. In skeletal muscles a necrotic vasculitis was seen in two pigs at PID 14.

#### Experiment III

**Figure 7:**
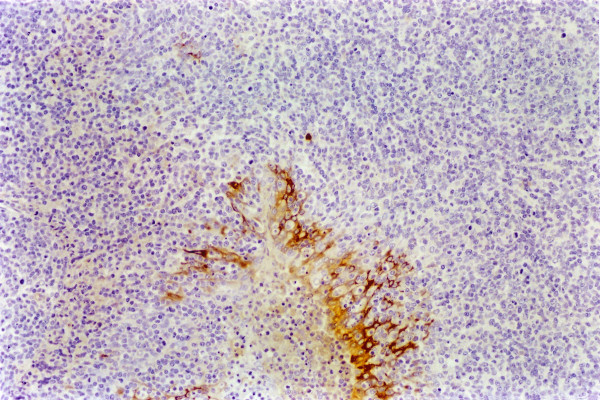
**Tonsil. Experiment III, PID 8. Positive immunohistochemical staining Tonsil**. Experiment III, PID 8. Immunoreactivity to WH 303 monoclonal antibody as a cytoplasmic rim in the crypt-epithelial cells. EnVision™ +HP mouse system. Magnification 540×.

In tonsils, mild changes were characterized by expanded crypts plugged with cellular debris and keratin. Positive immunostaining was observed from PID 5, in a few crypts/crypt epithelial cells of three pigs (Figure 7). In lymph nodes, neither atrophic changes nor haemorrhages were detected, but occasionally slight follicular and perifollicular hyperplasia were seen in most of the pigs from PID 2. From PID 5, very mild necrotic lesions of variable degree were observed in the lymph follicles of four animals. Positive immunostaining in macrophages was observed in one submandibular and one mesenteric lymph node at PID 7 and 8, respectively. No changes were noted in the spleens. In kidneys, a very mild focal interstitial nephritis with mononuclear cells was seen in about the half of the animals throughout the observation period. In addition, a focal mononuclear perivasculitis in the medulla was detected in two pigs at 60 hours, respective four days after infection. Specific immunostaining was not detected. In brain tissue, necrotic lesions were not seen, only swelling of the endothelial cells of the small vessels was observed. Specific immunostaining was not detected.

A general observation was that the microscopic changes in Experiments I and II became progressively more severe. In contrast, the changes seen in Experiment III remained fairly homogenous throughout the observation period.

In the uninfected control pig, histopathological changes and positive immunoreactivity was not observed. The sections of infected animals showed negative results when instead of specific antibody, 2% BSA was applied.

**Figure 8:**
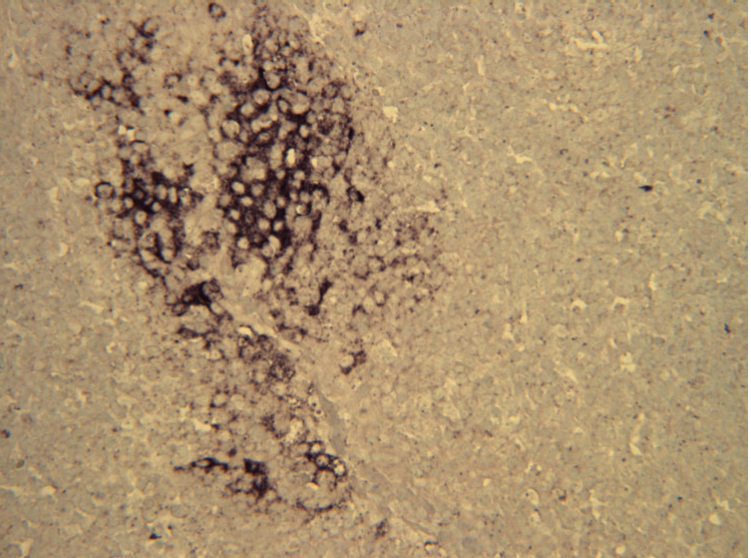
**Tonsil. Experiment II, PID 4. *In situ *hybridisation**. Tonsil. Experiment II, PID 4. Intense hybridisation signal for CSFV nucleic acid in the cytoplasm of tonsillar crypt epithelial cells. *In situ *hybridisation; DIG-labelled riboprobe. Magnification 675×.

The presence of CSFV nucleic acid was demonstrated by a pilot *in situ *hybridisation in various organs in all the three experiments. In experiment I the tonsils gave rather strong positive signals (4–10 foci/section) as early as 60 hours post infection. On PID 8 the distribution of viral nucleic acids was wide, strong hybridisation signals (> 10 foci/section) were seen in the tonsils, spleen, kidneys, various lymph nodes and in the lungs. In experiment II also the tonsils became first positive, but much later then in Experiment I. The first positive results in the tonsils were seen here 4 days post infection. Subsequently, 5 days post infection the spleen became positive; while on PID 8 the tonsils, spleen, kidneys, lymph nodes and lungs harboured viral nucleic acids. By reading the hybridisation assay, fewer foci were seen then in Experiment I (1–3 foci per section). The positive nucleic acid hybridisation signals in Experiment III were fewer (1–3 foci per section) and restricted to the tonsils and lymph nodes. The signals were observed between PIDs 3 to 8 in this group. The hybridisation signals were observed in the cytoplasm of the epithelial (Figure 8), mononuclear and reticular cells. When using the probe on the sections of the uninfected animals or the BHV-5 specific probe on the sections of the infected pigs, no hybridisation signal was observed.

## Discussion

Although CSF is registered as one of the most important Transboundary Animal Diseases (TADs), notifiable to OIE, the regular re-occurrence of the outbreaks in various regions of the world indicates that many questions are still poorly answered concerning the biology of this devastating disease. One of the problems is that CSF has an increasing tendency to appear and re-appear in a clinically very mild or in a completely unapparent form. By being unnoticed for a time, such mild infections may spread to large populations of pigs, causing serious epizootiological and economic consequences.

Considering the varying clinical manifestations and the observed diagnostic problems, further research has to be conducted on comparative pathology of CSFV, with special regard to the emerging new viral variants of very mild pathogenicity, causing very weak or completely unapparent clinical symptoms, which can easily be overlooked in the field. Regarding these requirements, we were conducting here *in vivo *studies on three groups of experimentally infected pigs, in order to compare the effects of viruses of varying virulence, which may occur in the field either as single or as multiple infections.

Other research groups reported on comparative *in vivo *analysis of CSFV strains [8,9,13] but none of the previous investigations provided such a comprehensive analysis of various virulence variants as the present study. The comparative analysis, performed on a large number of pigs under harmonised experimental conditions, is providing further data and demonstration material on the pathogenesis of CSF. In addition, the data are useful for the improvement of CSF diagnosis, with special regard to cases when the virus replication results only in the mild or inapparent clinical symptoms. Considering the high number of pigs (67 animals), it was preferable to divide the tasks and to perform the experiments at two partner laboratories in parallel, under harmonised experimental conditions. The same age groups of pigs were infected and sampled by using standardised procedures. The evaluation methods were also harmonised (like gross pathology, virus isolation) and all samples were collected for testing in a single laboratory by the same researcher (like histopathology, IHC and ISH).

The clinical signs, which are important for the early detection of the new cases of CSF infections in the field and for early warning [4] varied remarkably in the three groups. The febrile reactions were very marked in group I, since high fever was recorded already at PID 1 and it lasted throughout the experiment. Group II showed a later occurring and milder febrile reaction, while group III reacted only with a slightly elevated temperature, which did not show a marked profile. Inappetence also varied strongly; in groups I and II reduced appetite was observed from PID 1, respectively from PID 11, while in group III loss of appetite was not observed. Nervous symptoms also appeared with great variations: group I developed serious signs of the involvement of nervous system, while the other two groups remained symptomless, except for three pigs in group II, which developed slight ataxia one day before death.

Concerning viraemia, the differences were also very clear among the groups. In groups I and II the viral invasion in the blood circulation was recorded from PID 2, respectively PID 5, while group III developed no measurable viraemia. It is worth to note that the frequency of viraemia showed strong variations: in group I all animals became viraemic (from PID 4), while in group II only one, indicating that the virulence variants had various capacities of *in vivo *viral replication and invasion.

It is a known fact that the moderate or the low virulence variants of CSFV frequently cause very mild and/or unapparent clinical symptoms, which are accompanied by a restricted *in vivo *viral replication and invasion [13,14]. This phenomenon was clearly demonstrated and confirmed in the present experiments. Concerning epidemiology and early diagnosis of CSF, group II is the most interesting in our present studies. Based on our previous experiments and on the observations of other groups, we supposed that group II requires special attention (see notes above). This is the reason that group II was tested not only by the same methods as the two other groups, but also by DIF, in order to investigate the tissue distribution of the moderately virulent virus by as many means as possible. This selected group showed that a moderately virulent virus is able to cause infection without the development of any apparent clinical response. Simultaneously, the viral replication and invasion showed a restricted tendency in animals infected with the moderately virulent virus. In this group the number of diseased animals was lower than in group I and only three pigs died. Noteworthy, the development of the CSF varied remarkably in group II between the individual animals, ranging from a symptomless infection to typical, fatal cases. One can conclude that the lack of clinical symptoms and of detectable virus in the blood circulation in a number of animals may create serious problems in the early detection of an outbreak, caused by such variants of the virus. Due to the lack of clinical signs of diagnostic importance, special attention has to be paid to detect successfully and immediately such cases of CSFV infection in the field.

By comparing the gross pathological changes, in group I a distinct swelling of the submandibular lymph nodes was seen as early as PID 2. From PID 4 all the infected animals showed typical CSF lesions. In contrast, group II exhibited gross pathological signs only from PID 8, such as swelling and haemorrhages of lymph nodes as well as haemorrhages of kidneys. The wide range of typical pathological changes occurred in this group only from PID 12. In group III the lack of pathological changes indicated the attenuated character of the virus. One can conclude that the gross pathological findings were in good correlation with the clinical pictures observed in the three groups.

The histopathological examinations revealed marked differences among the three groups, which agreed with the clinical and gross pathological findings. The microscopic lesions included vascular changes and necrosis of lymphocytes, which were observed in all the three infected groups. The changes were more frequent and severe in the first two groups. Encephalitis, another major histopathological lesion, was seen only in groups I and II. Compared to group I, in group II the above mentioned lesions developed 5–6 days later, and remarkably, they became more severe at the termination of the experiment. These findings indicate that the highly and the moderately virulent viruses have rather similar capacities to induce histopathological changes, but in the case of the latter, these changes develop after a longer incubation period. These days, when the animals are already CSFV infected but neither clinical signs, nor histopathological changes are yet observed, creates an important risk-period in the safe early diagnosis of CSF.

By evaluating the findings in comparative histopathology, it has to be stated that in groups I and II the microscopic changes became progressively more severe during the development of the disease, in contrast to the lesions seen in group III, which remained fairly unaltered. These are factors, which should be considered in the comparative pathology and diagnosis of CSF.

Immunohistochemistry, in correlation to histopathology, also revealed marked differences among the groups. For example, group I showed necrotic changes of the lymphoid cells in the tonsils as early as PID 1 and the viral antigens became detectable from PID 2. In contrast, group II showed lymphoid cell necrosis only from PID 6. It is worth to note that the viral antigen appeared in group II prior to necrosis, since IHC became positive already from PID 4. The observed differences indicate the possibility of a very prompt and destructive viral replication in group I, leading to early cellular damage appearing very rapidly, before the detection of the virus by IHC. In contrast, in group II IHC revealed the signs of viral replication before the appearance of necrotic alterations. This indicates characteristic differences in the replication features and in the pathobiology of these two virulence variants. The phenomenon has diagnostic importance, since it illustrates that in the case of moderately virulent viruses, IHC is detecting the viral infection earlier, compared to the histological examinations. This is in accordance with the observations of Kamolsiriprichaiporn et al. [8]. Thus, the viral antigen demonstration is very important in the early detection of CSFV infections, especially in cases caused by moderately virulent viruses. The importance of diagnostic IHC is further emphasized by the observation that this test gave positive results very long time (seven days in our case) before the appearance of the clinical symptoms.

In contrast to the other two groups, no necrotic changes of the tonsils were detected in group III, confirming the very low virulent or avirulent character of the vaccine virus. However, viral antigen was detected in the tonsils by IHC, indicating viral replication. When discussing the virulence level of the vaccine strain, it is noteworthy that in the lymph nodes even this virus was able to induce necrotic changes from PID 5. These changes were presumably connected to viral replication, since IHC revealed the presence of viral antigens from PID 5 in the lymph nodes.

The results of virus isolation were in accordance with the tendency of IHC, since in group I the virus was detected in tonsils, spleen and lymph nodes as early as PID 2, while in group II it was isolated first on PID 4 from tonsils and lymph nodes. In group III the results of virus isolation agreed with the findings of IHC, since the vaccine strain was demonstrated in the lymphoid tissues between PID 3 and 7. However, it is remarkable that the replication of the virus was demonstrated by this test in not more than three animals. One can hypothesize that this was either a technical problem, or the amount of vaccine virus was so low that it was under the level of the detection capacity of the VI test in quite a number of animals.

As further tools of direct virus detection, DIF and IHC proved to be complementary methods to the "golden standard" of VI [9]. In our experiments IHC revealed the presence of the virus in the tonsils and lymph nodes in group I as early as PIH 60. It is interesting that in striated muscles and heart the virus was detected by VI, but not by IHC. One can speculate that this might be due to two basic reasons: i) the sensitivity of IHC is lower; ii) the virus is transported to these organs by blood, due to viraemia, which is detected by VI but not localised by the IHC method [15]. In agreement with the previous results, DIF and IHC detected the virus in group II from PID 4. Similarly to VI, the virus was detected by DIF and IHC in the tonsils and in addition, IHC gave positive results also in the spleen and lungs. It is noteworthy, that nervous tissue showed both histopathological lesions and the presence of viral antigen in Experiment I, while in Experiment II in spite of severe histopathological changes, CSFV antigen was not detected. To explain this peculiar phenomenon, one can speculate that: i) early cell damage may occur already at initial stage of viral replication, when the viral load is still low; ii) immune-mediated reactions may play role [16]. Concerning group III, the results of IHC indicated some virus replication between PIDs 5 and 8 in the tonsils and in the lymph nodes, but similarly to VI, only in three animals. This finding confirms that the vaccine strain replicates in the lymphoid tissues; while the amount of replicating virus is presumably low.

The quantitative tendencies of the virus replication will be investigated by the real-time PCR assays of our laboratories [17] in the forthcoming experiments Since the viral nucleic acid detection and quantification by PCR and by other means of molecular diagnosis has various approaches and variants, the involvement of those assays would turn the present paper extremely long, complicated and multidisciplinary. Thus, herewith we focused on the comparison of morphology-associated descriptions and diagnostic approaches; compared to the golden standard of virus isolation, while the PCR investigations will be reported and discussed in separate articles.

The present results show that the *in situ *hybridisation technique, developed in this study, is a useful tool for the detection of CSFV in formalin fixed, paraffin embedded tissue samples. Similarly to the observation of Choi and Chae [18]*in situ *hybridisation assays provide sensitive means for studying the pathogenesis of acute and chronic CSFV infections.

The parallel studies on the three experimental groups allowed not only the comparison of clinical, pathological and virological parameters, but also the estimation of further aspects of disease development. It is clear that group I represented the case of rapidly developing, fatal CSF. However, group II, which developed an initially more subtle, milder disease, revealed many aspects, which might be useful considering the recent epizootiological situations in swine populations. An interesting observation in this group is that the number of cells immunopositive for the viral antigen had a tendency to decline slightly during the course of virus infection. Similar tendency has been reported in case of BVDV [19]. Concerning CSFV, Sánchez-Gordón et al. [20] have observed a similar decline in the tonsils. The intensity of the phenomenon varied in various experiments and the authors hypothesize that the differences might have been due to the timing of virus spread or differences in the local immune responses [20].

## Conclusion

The *in vivo *studies and the accompanied diagnostic approaches provided useful data on the comparative pathology of three virulence variants of CSFV. The experiments confirmed the previous expectations that the three variants represent various levels of pathogenicity. Data have been obtained concerning comparative aspects of clinical manifestations, development of pathological signs and tissue distribution of CSFV variants. These data have practical importance when discussing the pathobiology of classical swine fever in the host species. The observations are useful for the early diagnosis of classical swine fever, with special regard to the detection and identification of the very mild or inapparent clinical manifestations. The present study demonstrates that in the case of the highly and moderately virulent virus variants the virulence does not affect the pattern of the spread in a pig, but influences the onset, intensity, duration and outcome of the disease. As far as diagnostic tools are concerned, IHC provides useful means of early virus detection and it indicates the localisation of the virus spread in tissues, supporting the determination of the pathogenicity levels of newly emerging viruses.

## Competing interests

The authors declare that they have no competing interests.

## Authors' contributions

KB performed the histopathological, immunohistochemical and *in situ *hybridisation studies, participated in the evaluation and summarizing of the findings and wrote the draft of the manuscript. FK applied for funding of the project, participated in the design of the study and performed the second animal experiment inclusive virus isolation as well as participated in the evaluation of the findings and had a major impact on the manuscript. HV participated in the design of the study and performed and evaluated the second animal experiment inclusive virus isolation. CM participated in the design and performing of the *in situ *hybridisation study, evaluated the findings and influenced the manuscript. FF participated in performing the second and third animal experiments inclusive virus isolation and evaluating the results. GMDM participated in the design of the study, participated in performing the second and third animal experiments inclusive virus isolation, evaluated the findings and influenced the manuscript. SB applied for funding of the project, participated in the design of the study and had a major impact on the manuscript. All authors read and approved the final manuscript.
